# Application of Surface Protective Coating to Enhance Environment-Withstanding Property of the MEMS 2D Wind Direction and Wind Speed Sensor

**DOI:** 10.3390/s17092152

**Published:** 2017-09-19

**Authors:** Kyu-Sik Shin, Dae-Sung Lee, Sang-Woo Song, Jae Pil Jung

**Affiliations:** 1 Smart Sensor Research Center, Korea Electronics Technology Institute, Seongnam 13509, Korea; neokarion@ketir.re.kr (K.-S.S.); leeds@keti.re.kr (D.-S.L.); kano2@keti.re.kr (S.-W.S.); 2 Department of Material Science & Engineering, University of Seoul, Seoul 02504, Korea

**Keywords:** MEMS, wind sensor, protective coating

## Abstract

In this study, a microelectromechanical system (MEMS) two-dimensional (2D) wind direction and wind speed sensor consisting of a square heating source and four thermopiles was manufactured using the heat detection method. The heating source and thermopiles of the manufactured sensor must be exposed to air to detect wind speed and wind direction. Therefore, there are concerns that the sensor could be contaminated by deposition or adhesion of dust, sandy dust, snow, rain, and so forth, in the air, and that the membrane may be damaged by physical shock. Hence, there was a need to protect the heating source, thermopiles, and the membrane from environmental and physical shock. The upper protective coating to protect both the heating source and thermopiles and the lower protective coating to protect the membrane were formed by using high-molecular substances such as SU-8, Teflon and polyimide (PI). The sensor characteristics with the applied protective coatings were evaluated.

## 1. Introduction

Ever since the microelectromechanical system (MEMS) was established in the 1980s, the advantages of MEMS technology, that is, small size, mass production, low power consumption, accuracy, and so forth, have been widely acknowledged. Recently, due to these advantages, many MEMS sensors are being applied in IoT (Internet of Things) technology. The most widely used sensors in this technology are pressure, gyro, acceleration, and temperature sensors. Many efforts have been made by scientists to develop MEMS wind direction and speed sensors, but commercialized products have not yet been released. Thermal-type MEMS wind direction and speed sensors have been developed, consisting of a heating source and thermopiles on a membrane. However, the detection area of these sensors was exposed in air to sense the wind direction and wind speed, and hence to protect them from electrical, environmental, and physical shock, a protective layer was required [1,2,3].

Typically, materials such as parylene, silicon oxide and nitride thin film have been used as sensor protection layers. There are three types of parylene (-*N*, -*C*, -*AF4*); parylene-*N* and parylene-*C* have hydrophilic properties, and parylene-*AF4* has hydrophobic properties. When the surface of the protective layer is hydrophobic, surface contaminants can be removed by the lotus effect. However, the common method of parylene coating is to use vacuum equipment in a chemical vapor deposition (CVD) method, which leads to an increase in the cost of product development. The wetting angles of silicon oxide and silicon nitride are in the range of 7–29°. The hydrophilic characteristics of silicon oxide and silicon nitride have been previously shown [3,4,5,6,7,8].

In this paper, a protective coating was applied to the surface of the sensor by using high-molecular substances such as SU-8 (SU-8 2000.5, MicroChem, Westborough, MA, USA), Teflon (Teflon AF 1601S, Dupont, Wilmington, DE, USA) and polyimide (PI, HD-4000, HD MicroSystems, Parlin, NJ, USA) to protect the heating source, thermopiles, and membrane for maintaining or improving the sensor functions, and the various sensing characteristics were evaluated.

## 2. Materials and Methods

### 2.1. Driving Principle

**Figure 1:**
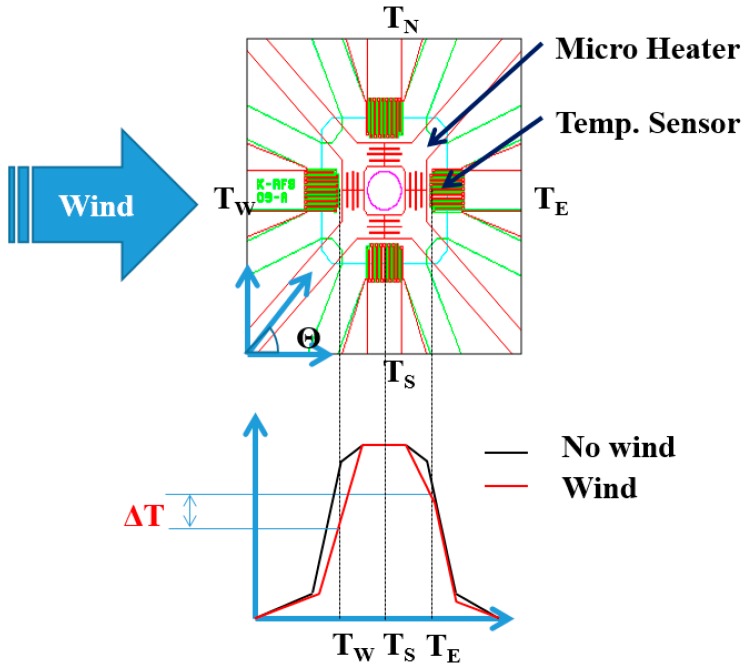
Driving principle of the microelectromechanical system (MEMS) two-dimensional (2D) wind direction and wind speed sensor.

The manufactured MEMS two-dimensional (2D) wind direction and wind speed sensor used the thermal detection method, consisting of a square heating source at the center of a membrane and thermopiles arranged facing four different directions, east, west, south or north, around the heating source. (Figure 1).

The driving principle of the MEMS 2D wind direction and wind speed sensor is as follows. When the direction of the wind blows from west to east, the temperature of the west side of the sensor is lowered, and the temperature of the east side rises. As a result, there is a temperature difference between the east and west sides of the sensor. On the other hand, the south and north sides have the same reducing temperature rate and hence there is no temperature difference between the south and north sides of the sensor. The thermopiles in each direction generate an output voltage according to the temperature change.

The resultant wind speed can be given by the formula:

(1) $$\sqrt{\Delta V_{EW}{}^{2}+\Delta V_{SN}{}^{2}},$$

Δ*V_EW_*: voltage difference of the east and west side thermopiles on the sensor.
Δ*V_SN_*: voltage difference of the south and north side thermopiles on the sensor.

In addition, the wind direction at the same time can be calculated through the formula:

(2) $$\theta =\tan^{-1}\frac{\Delta V_{EW}}{\Delta V_{SN}},$$

### 2.2. Manufacturing Process

**Figure 2:**
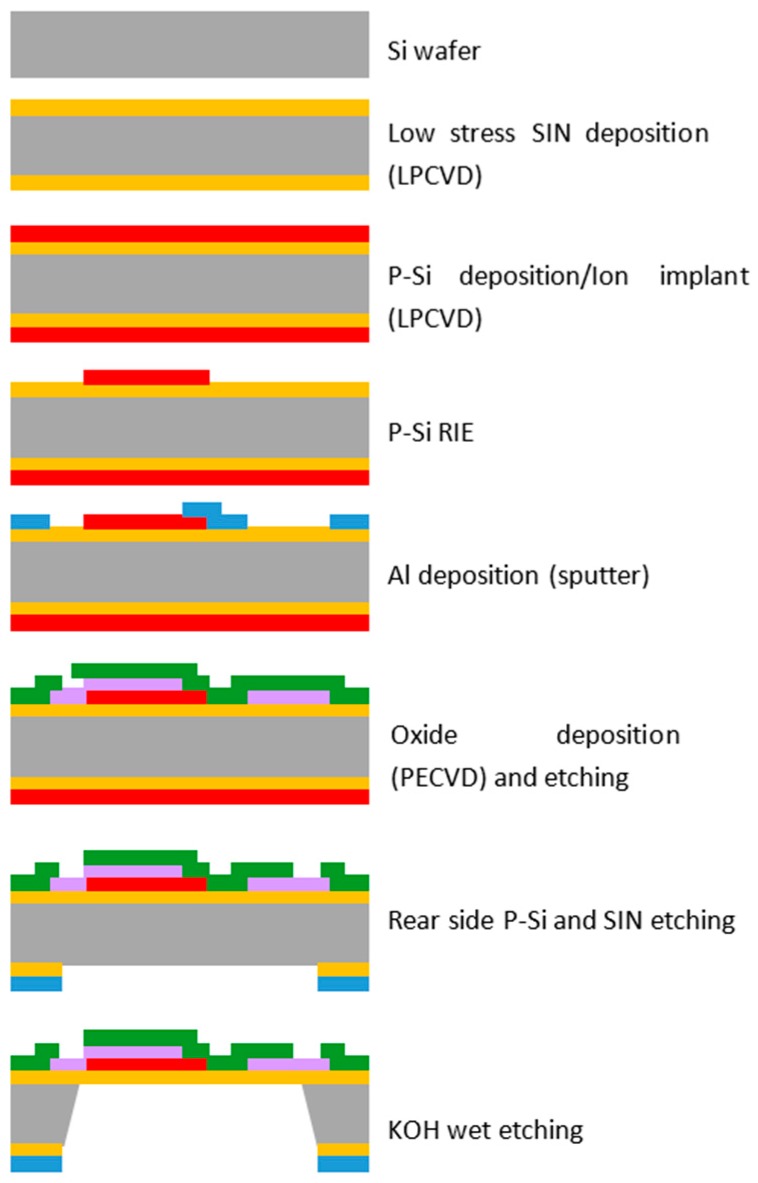
Manufacturing process of the MEMS 2D wind direction and wind speed sensor.

**Figure 3:**
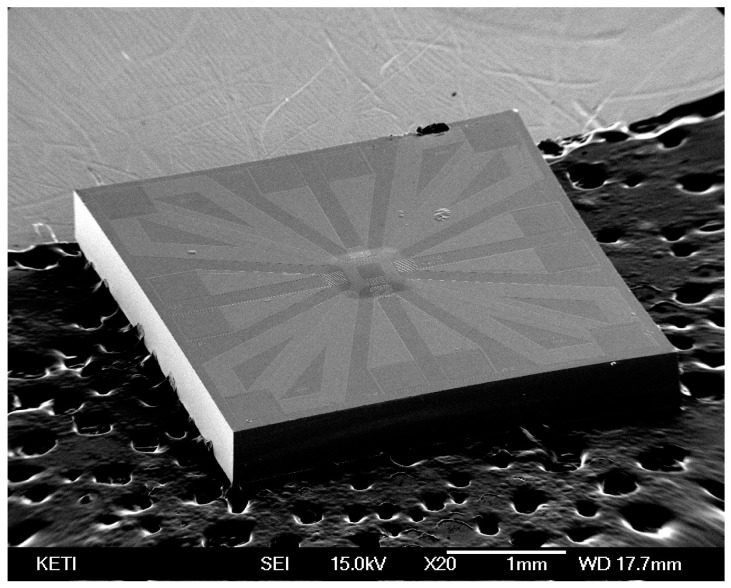
Appearance of the manufactured MEMS 2D wind direction and wind speed sensor chips.

A MEMS 2D wind direction and wind speed sensor with a size of 3.5 mm × 3.5 mm was manufactured by the following process. Low-stress silicon nitride with a size of 1 µm was deposited on the silicon substrate (4 inch, 525 um, DSP, LG Siltron, Gumi, Korea) with a thickness of 500 µm using low-pressure chemical vapor deposition (LPCVD). Polysilicon was then deposited on the silicon nitride up to a thickness of 0.2 µm by LPCVD (Sungjin Semitech, Cheongju, Korea). Patterning was performed to form a heating source on top of the surface. Subsequently, aluminum was sputtered onto the surface with a thickness of 0.5 µm to form thermopiles and the electrode. A passivation layer of silicon dioxide to protect the heating source was formed using plasma-enhanced chemical vapor deposition (PECVD, NEXSO, Hwaseong, Korea). Finally, a membrane was formed by a dry etching process. (Figure 2 and Figure 3).

### 2.3. Evaluation of Basic Features (Measurement Results of Wind Speed, Wind Direction)

**Figure 4:**
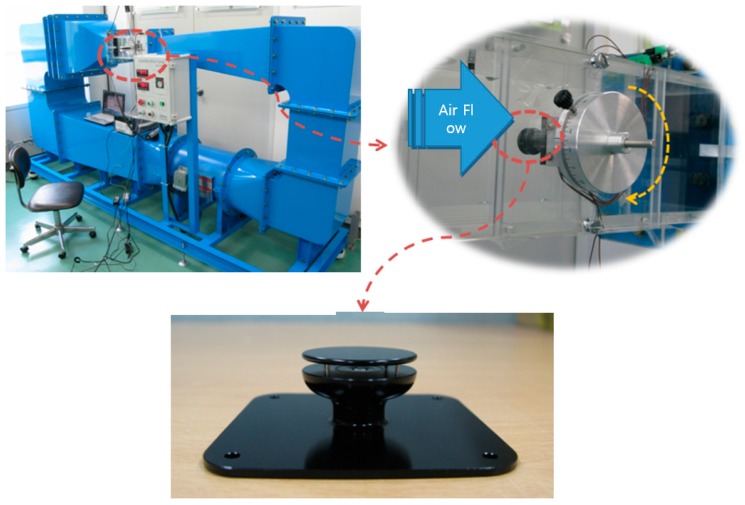
A wind tunnel for measuring the manufactured MEMS 2D wind direction and wind speed sensor.

Figure 4 shows a wind tunnel used to test the wind speed and wind direction measurements of the manufactured MEMS 2D wind direction and wind speed sensor. The wind tunnel was composed of a jig which could be used to adjust the wind speed to approximately 45 m/s, as well as the wind direction by turning the sensor to an angle of up to 360°.

**Figure 5:**
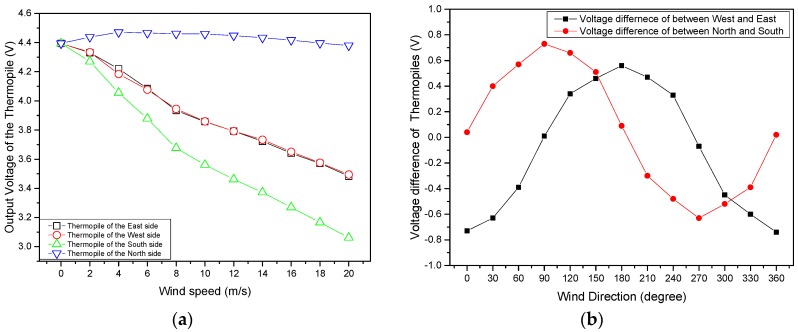
Measured output voltages of thermopiles depending on wind speed and wind direction. (**a**) The output voltage with wind speed (the wind direction is from south to north); (**b**) Voltage difference for west-east and north-south with wind direction (the wind direction at zero degrees is from north to south).

Wind speed and wind direction were measured to identify the sensitivity of the manufactured MEMS 2D wind direction and wind speed sensor. As shown in Figure 5a, the output voltage of the thermopile was reduced depending on the increase in the wind speed. It was found that the sensitivity of the sensor was approximately 0.07 V·(m/s)^−1^. A comparison of the velocity range and sensitivities for relevant 2D wind sensors is given in Table 1.

The voltage difference was generated as the cosine angle between east and west, and the sine angle between south and north at a constant wind speed of 10 m/s (Figure 5b).

### 2.4. Presentation of Problems and Solution

Both the heating source and thermopiles of the manufactured MEMS 2D wind direction and wind speed sensor have structures exposed to the atmosphere in order to detect wind speed and wind direction. Hence, the detection ability of the sensor is reduced due to contamination by deposition or cohesion of dusts, sandy dusts, snows, rains, and so on, in the air. In addition, a thin membrane with a thickness of 1 µm may be damaged by physical shock due to the pressure difference between the top and bottom of the membrane caused by wind flow.

**Figure 6:**
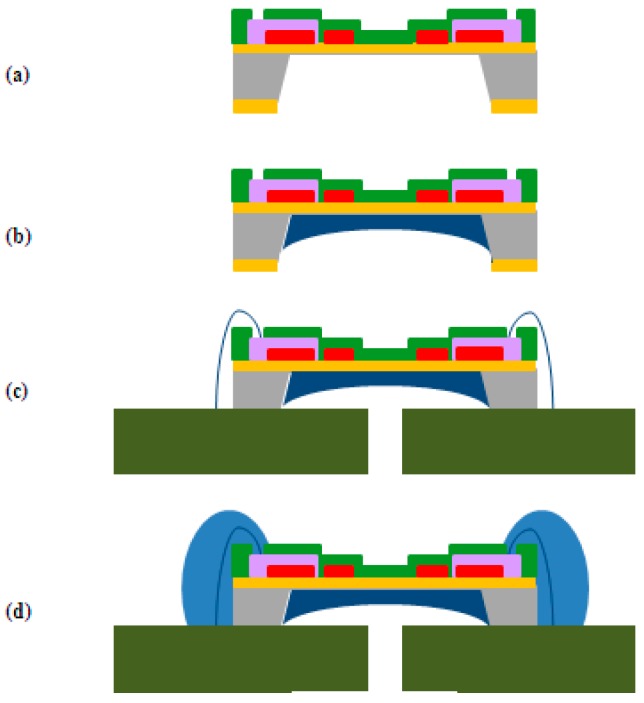

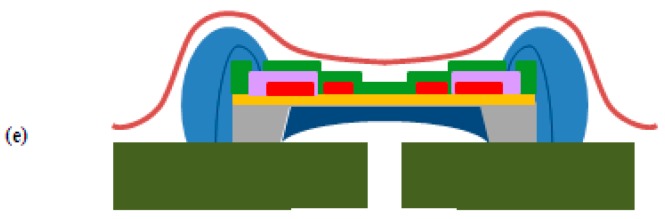
Flowchart for designing protective coatings. (**a**) KOH wet etching; (**b**) Polymer back side molding; (**c**) Chip attach (PCB) and wire bonding; (**d**) Epoxy molding; (**e**) Surface coating.

A protective coating for the heating source, thermopiles, and membrane was designed using high-molecular substances (SU-8, Teflon, PI), which generally allow semiconductor processes so that the coating could be applied to the manufactured MEMS 2D wind direction and wind speed sensor. Figure 6 shows a flowchart of a surface protective coating designed to protect the heating source, thermopiles, and the membrane. The upper surface protective coating of the membrane was to protect both the heating source and thermopiles from environmental dust, sandy dust, snow, rain, and so forth, in the air that can be deposited or adhered, while the lower surface protective coating of the membrane was to prevent membrane damage from physical shock.

## 3. Results

### 3.1. Qualification Test

#### 3.1.1. Wettability and Sensitivity of Upper Protective Coating

The upper protective coating of the membrane must have excellent hydrophobicity so that dust, sandy dust, snow, rain, and so on, in the air will not be deposited or adhered on the sensor surface, and must not be easily separated from the sensor surface.

**Figure 7:**
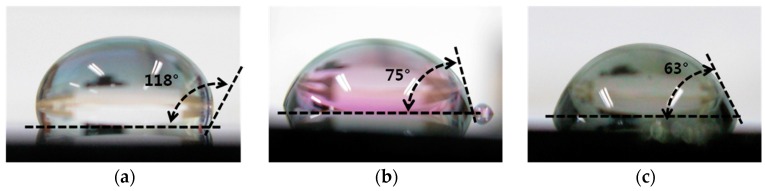
Photographs measuring contact angles of: (**a**) Teflon; (**b**) SU-8; and (**c**) polyimide (PI).

The contact angle was measured after a coating film was formed and a deionized water drop (diameter; 1 cm) was poured onto the silicon wafer, where a layer of silicon oxide (0.2 µm) had been deposited on the selected high molecular substance using a spin coating method, and the adhesive strength of the coating film was compared using a tape test. The Teflon coating was found to show excellent hydrophobicity, with a contact angle of 118°, compared to the other two high molecular substances (SU-8: 75°, PI: 63°). It was, however, demonstrated that the Teflon coating has a weak adhesive strength as it was separated from the wafer with tape. There is thus a need to supplement the adhesive strength of the Teflon coating. SU-8 was used as an adhesive layer between the sensor surface and the Teflon coating to enhance the adhesive strength of the Teflon coating. (Figure 7).

First, an SU-8 coating layer with a thickness of 0.5 µm was applied through a heat treatment for 60 s, at 373 K on a hot plate after spin coating (3000 rpm, 30 s). Subsequently, Teflon, with a thickness of 1 µm was coated on the surface of this SU-8 through heat treatment for 5 min, at 373 K on a hot plate after spin coating (1000 rpm, 20 s).

**Figure 8:**
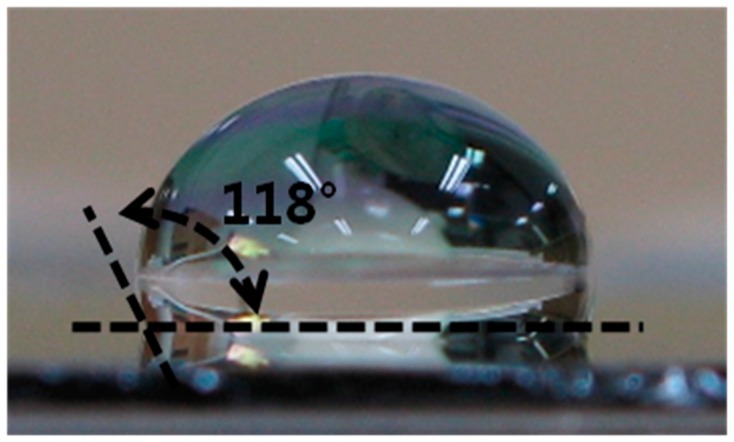
The contact angle of the upper protective coating with 2-Layer (Teflon/SU-8).

The contact angle of the two-layered upper protective coating was 118°, as shown in Figure 8, and it was found that the adhesive strength of this coating was also excellent, as confirmed from the result of tape testing.

**Figure 9:**
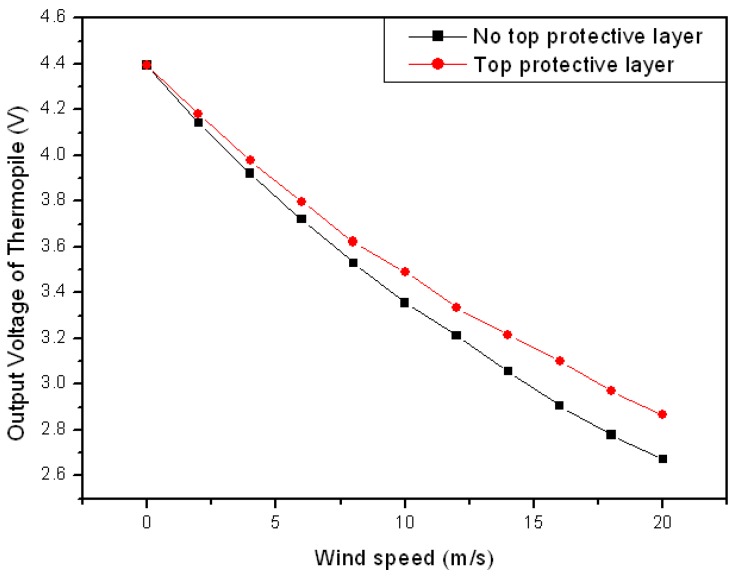
Wind speed measurements of the sensor with and without the two-layered upper protective coating.

Figure 9 shows a graph comparing the wind speeds measured by the sensor with and without the two-layered upper protective coating. The sensitivity of the two-layered upper protective coating was reduced by 14% (0.2 V voltage difference at 20 m/s) compared to the sensitivity without the upper protective coating (Table 2).

#### 3.1.2. Evaluation of the Lower Protective Coating

The lower protective coating was applied to protect the sensor membrane, which is easily damaged by external physical shock. Teflon and SU-8 (Figure 8) were used as the upper protective layer. Following this, the rear side of the membrane was filled using SU-8, Teflon, or PI as lower protective layers.

The viscosity of PI was found to be higher than that of the other two substances, and so it was diluted with *N*-methyl-2-pyrrolidone (solvent) at a mass ratio of 1/2:1 before use so that it could more easily fill the membrane. Response times and output voltages were measured through heat shock tests (temperature cycle tests) after the three substances were applied as a lower protective coating.

##### Heat Shock Test

**Figure 10:**
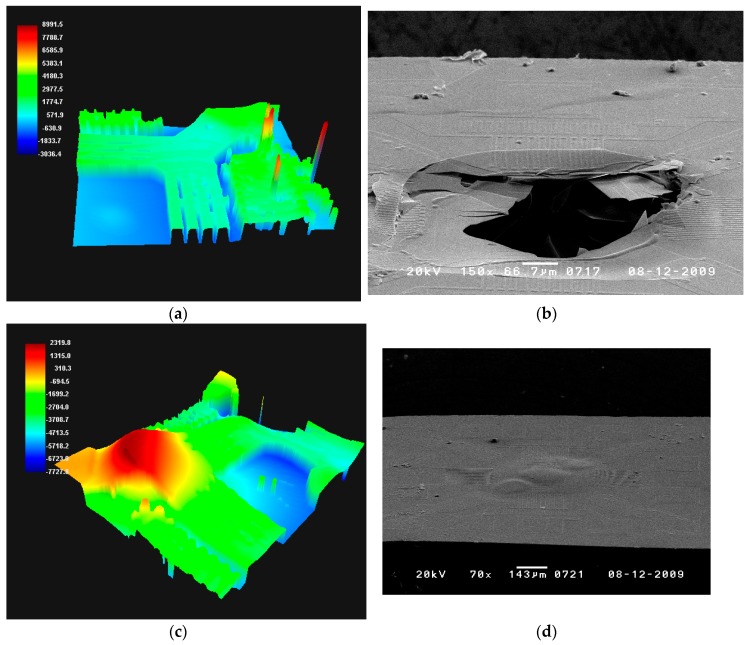

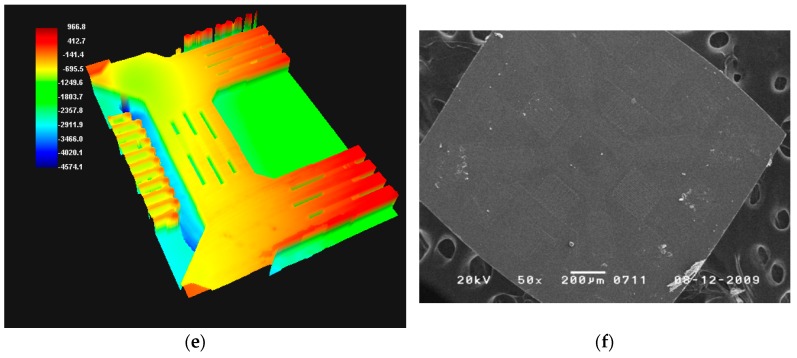
Three-dimensional (3D) profile result and scanning electron microscopy (SEM) images of (**a**,**b**) Teflon, (**c**,**d**) SU-8, and (**e**,**f**) PI.

A heat shock test was performed to evaluate the resistance of the membrane against fast temperature changes of the sensor with a lower protective coating. Table 3 shows the regulations of the heat shock tests. According to these regulations, a total of 100 cycles were applied; in each cycle, the sensor was at a temperature of 353 K (and 218 K), which was maintained for 10 min, and after that the temperature of the sensor was immediately changed to 218 K (and 353 K) (temperatures for condition A) (Table 3, Figure 10).

The membrane status was analyzed using scanning electron microscopy (SEM) and 3D profiling (PWM-T250). The membrane with the SU-8 coating was deformed as it partially expanded at the heating source as a result of the heat shock, and the membrane with the Teflon coating was damaged. It was determined that the membrane was damaged according to the thermal expansion coefficients of both the membrane and Teflon coating shown in Table 4. On the other hand, the membrane with a PI coating was not deformed or damaged, showing a relatively stable status. It is thus expected that application of PI as a lower protective coating will prevent any deformation or damage of the membrane which may be caused by a sudden external change of temperature.

##### Comparison between Response Time and Output Voltage

**Figure 11:**
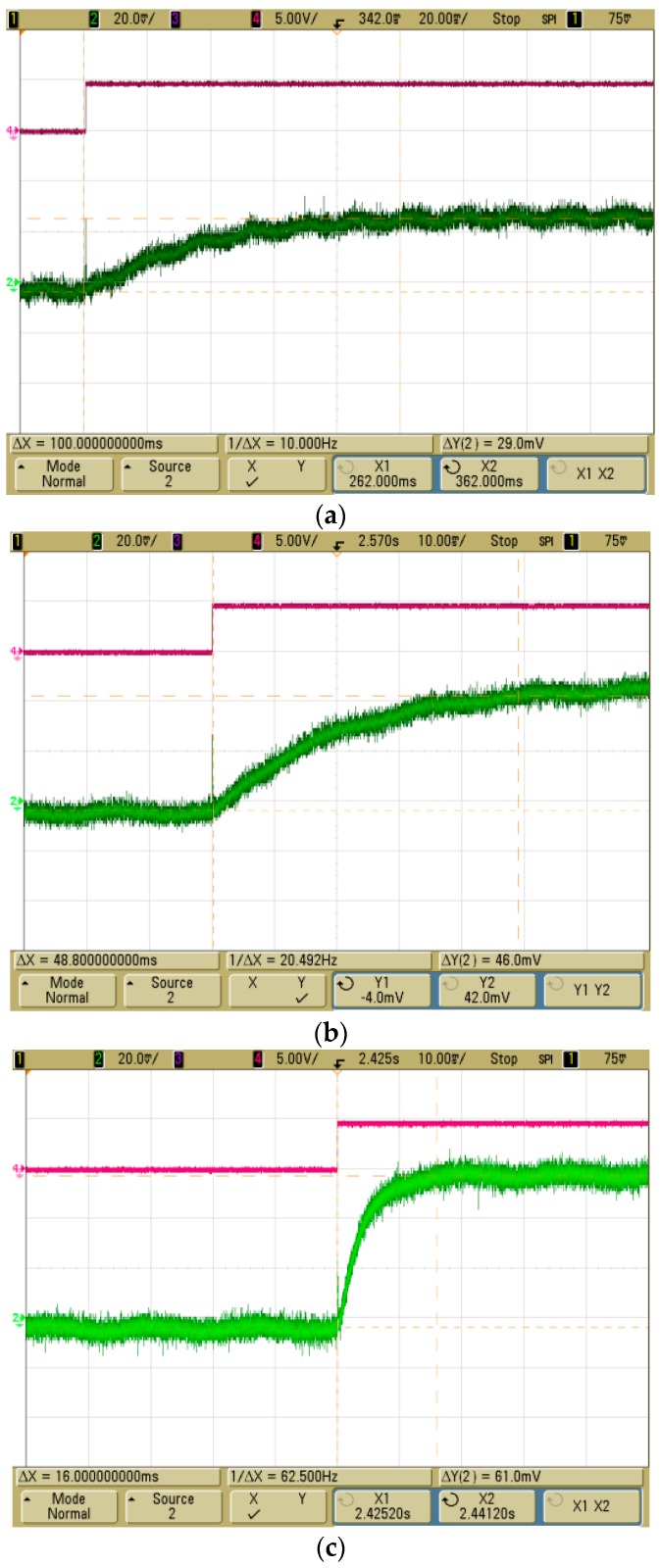
Comparison between response times and output voltages of thermopiles; (**a**) Teflon (100 ms, 29 mV), (**b**) SU-8 (48.8 ms, 46 mV), (**c**) PI (16 ms, 61 mV).

The response time and output values of the MEMS 2D wind direction and wind speed sensor, with different applied lower protective coatings, were measured for comparison. The response time and output voltage of a thermopile was measured using a MSO6034A (Agilent, Santa Clara, CA, USA) oscilloscope by applying a voltage of 5 V to the heating source at normal temperature after sealing the sensor to prevent external effects. The sensor with the PI coating showed a more rapid response time of 16 ms and a higher output voltage of 61 mV than the sensor with both SU coating and Teflon coating (Figure 11). The response time, output voltage and coating thickness are shown in Table 5. As the difference in thermal conductivity was small, the output voltage and response time were affected by the thickness of the coating.

##### Comparison between Heat Generation Characteristics of Input Voltage Depending on the Presence of a PI Membrane

The membrane status of the PI coating was similar to those of the SU-8 coating and the Teflon coating even for a sudden change of temperature, and showed the highest response time and highest output voltage; hence, this was selected as a lower protective coating. Subsequently, the heat generation characteristics of the heating source with and without the PI lower protective coating were compared.

**Figure 12:**
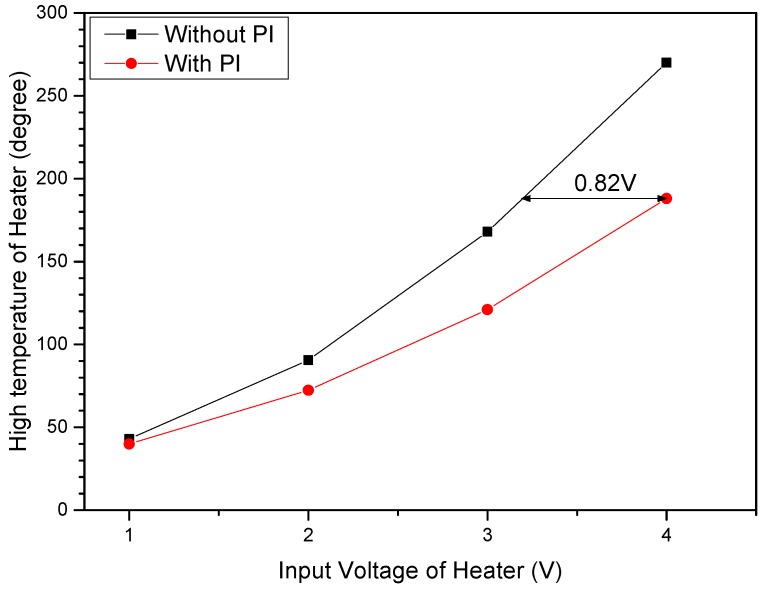
Comparison between heat generation characteristics of input voltage depending on the presence of a PI coating.

The temperature of the heating source with variation in the input voltage of heater was measured using a FLIR T250 thermography camera (FLIR, Wilsonville, OR, USA). The PI-coated sensor was need approximately 0.82 V at 183 °C as the low thermal conductivity of PI coating. (Figure 12).

##### Comparison of the Ability of the Membrane to Withstand Pressure with and without a PI Membrane

**Figure 13:**
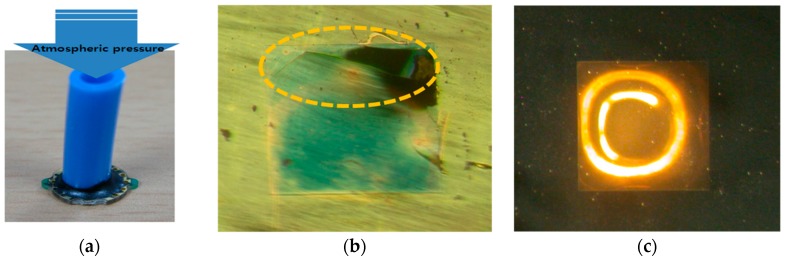
Comparison of the ability of a membrane to withstand pressure with and without the PI coating. (**a**) Sample made to measure ability to withstand pressure. (**b**) Without PI coating, membrane appears broken at 1.01 bar. (**c**) With PI coating, membrane appears unbroken even at 9 bar.

Atmospheric pressure from 1 bar (gauge) to 9 bar (gauge) was applied using a sensor tester (Ruska 7215, Ruska Instrument Corporation, Everett, WA, USA), which could measure and compare the ability of a membrane to withstand pressure with and without the PI coating. The membrane without the PI coating displayed damage at an atmospheric pressure of 1.01 bar, while the PI-coated membrane showed no damage even at 9 bar. It was thus concluded that the PI coating prevented damage to the membrane from physical shock. Figure 13 shows the physical appearance of the sample made for the pressure-withstanding measurements and the status of the membrane, with and without the coating, after the application of pressure.

##### Accelerated High Temperature/High Moisture Test for the MEMS 2D Wind Direction and Wind Speed Sensor after Application of Upper/Lower Protective Coatings

An accelerated high temperature/high moisture test to evaluate reliability was performed after applying the two-layered upper protective coating to the upper part of the manufactured MEMS 2D wind direction and wind speed sensor, and applying the SU-8 coating, the Teflon coating, or the PI coating as the lower protective coating.

**Figure 14:**
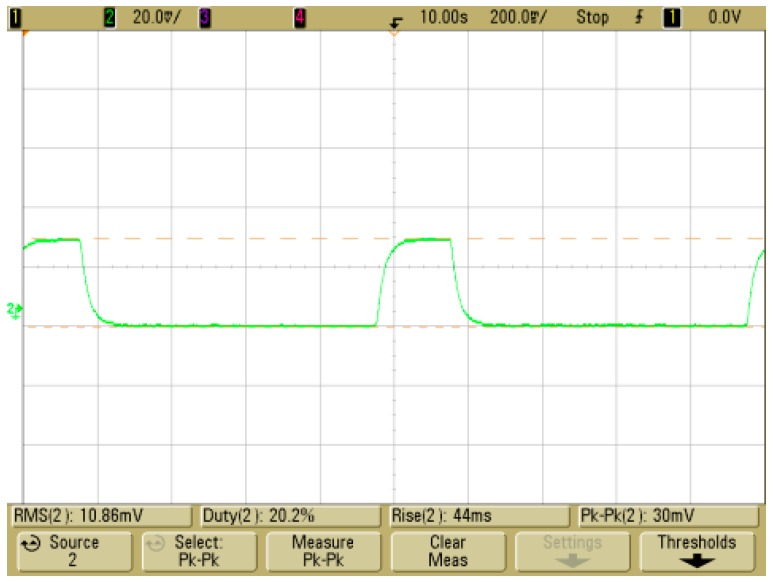
Accelerated high temperature/high moisture test result of the MEMS 2D wind direction and wind speed sensor applying both the two-layered upper protective coating and the PI lower protective coating.

The accelerated high temperature/high moisture test is a reliability test designed to accelerate erosion at the chip surface of the product. The sensor was left for 168 h at a temperature of 358 ± 2 K and a relative humidity of 85 ± 5%, established according to regulations of the IEC (International Electrotechnical commission) 60068-2-67. The temperature and humidity were then lowered to normal conditions, and the status of the membrane was then visually checked after 15 h. The membrane of the sensor with both SU-8 coating and Teflon coating was partially deformed and damaged, while the membrane of the sensor with the PI coating showed no change. The thermopile resistance of the sensor with the PI coating increased by 6% (11.636 to 12.303 kΩ) from the resistance before the test, and resistance of heating source increased by 100% (227.28 to 453.1 Ω) from the resistance before the test. The response time of the sensor with the PI coating was measured to be 44 ms, and the output voltage was measured as 30 mV (Figure 14).

## 4. Discussion

The MEMS 2D wind direction and wind speed sensor was manufactured using the heat detection method for the simultaneous measurement of wind speed and wind direction. This product consisted of a square heating source and four thermopiles.

The heating source, thermopiles, and membrane must be exposed to air in order to detect the wind speed and wind direction, and hence may be contaminated by dust, sandy dust, snow, rain, and so forth, in the air and could be damaged by physical shock. There is thus a need to protect the heating source, thermopiles, and membrane. In this paper, a protective coating was designed to protect the heating source, thermopiles, and membrane from contamination and damage by using high-molecular substances and the characteristics of the sensor with the applied protective coating were evaluated.

The upper protective coating of the membrane was manufactured in the form of a two-layered coating, with Teflon, which displays excellent hydrophobicity so can protect both the heating source and thermopiles, and SU-8 as an adhesive layer to complement the weak adhesive strength of Teflon. The sensitivity of the sensor with two-layered upper protective coating was reduced by 14% (a 0.2 V voltage difference at 20 m/s) compared to that without the upper protective coating.

The lower protective coating of the membrane was formed with high molecular substances applied below the membrane. In the case of PI coating, the membrane showed similar deformation behavior, even with a sudden change of temperature, to the coating with both SU-8 and Teflon, and showed a rapid response time of 16 ms and a high output voltage of 61 mV.

The membrane without the PI coating was damaged at an atmospheric pressure of 1.01 bar, while the PI-coated membrane was not damaged even at 9 bar.

Finally, an accelerated high temperature/high humidity test was performed for the MEMS 2D wind direction and wind speed sensor with both the two-layered upper protective coating and the lower protective coating of PI. The membrane of the sensor was not damaged or deformed after the test.

## 5. Conclusions

It is thus expected that the upper/lower protective coatings applied to the MEMS 2D wind direction and wind speed sensor will protect the heating source, thermopiles, and membrane from contamination by dust, sandy dust, snow, rain, and so on, in the air, as well as damage by physical shock, and therefore enable the sensor to withstand the environment.

## Figures and Tables

**Table 1:** Comparison of sensitivity for relevant 2D wind sensors.

Sensor	Velocity Range (m/s)	Sensitivity (V·(m/s)^−1^)
This work	0–20	0.07
Reference [2]	0–30	0.012
Reference [9]	0–2	0.133
Reference [10]	0–0.2	0.03
Reference [11]	0–5	0.00022
Reference [12]	0–1	0.0095
Reference [13]	0–0.1	0.041

**Table 2:** Comparison of specification conditions (no top protective layer/top protective layer).

Condition	Input Voltage (V)	Sensitivity (V·(m/s)^−1^)
No top protective layer	4.7	0.086
Top protective layer	4.7	0.076
Two-dimensional solid silicon thermal wind sensor	-	0.012

**Table 3:** The heat shock test standards.

Condition	Low Temperature (K)	High Temperature (K)
A	218 (+0/−10)	353 (+10,−0)
B	218 (+0/−10)	398 (+10,−0)
C	208 (+0/−10)	423 (+10,−0)
D	208 (+0/−10)	473 (+10,−2)
E	208 (+0/−10)	448 (+10,−0)
F	233 (+0/−10)	398 (+10,−0)
G	218 (+0/−10)	423 (+10,−0)

**Table 4:** Physical properties of the high molecular substances [14,15,16,17,18,19].

Property Type	Teflon 1601	SU-8 2000.5	PI HD-4100	Membrane
Modulus of elasticity (GPa)	1.6	2.0	3.3	166–297
Poisson ratio	0.39	0.22	0.34	0.23–0.28
Thermal conductivity (W/mK)	0.24	0.3	0.25	1.4–3.7
Thermal expansion coefficient (ppm/K)	260	52	35	10–43

**Table 5:** Comparison between response times and output voltages [14,15,16,17,18,19].

Property Type	Teflon 1601	SU-8 2000.5	PI HD-4100
Coating thickness (um)	5	3	1
Thermal conductivity (W/mK)	0.26	0.3	0.25
Response time (ms)	100	48.8	16
Output voltage (mV)	29	46	61
